# {6,6′-Dimeth­oxy-2,2′-[2,2-dimethyl­propane-1,3-diylbis(nitrilo­methyl­idyne)]diphenolato}nickel(II) 1.78-hydrate

**DOI:** 10.1107/S1600536809014500

**Published:** 2009-04-25

**Authors:** Chin Sing Yeap, Reza Kia, Hadi Kargar, Hoong-Kun Fun

**Affiliations:** a X-ray Crystallography Unit, School of Physics, Universiti Sains Malaysia, 11800 USM, Penang, Malaysia; b Department of Chemistry, School of Science, Payame Noor University (PNU), Ardakan, Yazd, Iran

## Abstract

In the title complex, [Ni(C_21_H_24_N_2_O_4_)]·1.78H_2_O, the Ni^II^ ion has a slightly distorted planar geometry, coordinated by the two N and two O atoms of the tetra­dentate Schiff base ligand, with a mean deviation of 0.272 Å from the NiN_2_O_2_ plane. The N and O donor atoms are mutually *cis*. The dihedral angle between two benzene rings of the ligand is 38.86 (8)°. There are also three solvent water mol­ecules, two of which lie across different crystallographic twofold rotation axes; one of these is partially occupied with a refined occupancy factor of 0.570 (7). The water mol­ecules are linked together as tetra­mers in *R*_2_^2^(8) ring motifs, which also connect two neighbouring mol­ecules of the complex through a network of O—H⋯O hydrogen bonds. The crystal structure is further stabilized by inter­molecular C—H⋯O and C—H⋯π inter­actions, which link neighbouring mol­ecules into extended chains along the *b* axis. Other inter­esting features of the crystal structure are the short inter­molecular C⋯C [3.204 (3)–3.365 (3) Å] and the C⋯O [3.199 (2)–3.205 (2) Å] contacts which are shorter than the sum of the van der Waals radii of these atoms.

## Related literature

For bond-length data, see: Allen *et al.* (1987 ▶). For related structures, see: Clark *et al.* (1968 ▶, 1969 ▶, 1970 ▶). For applications and bioactivity of Schiff base complexes, see: Elmali *et al.* (2000 ▶); Blower (1998 ▶); Granovski *et al.* (1993 ▶); Li & Chang (1991 ▶); Shahrokhian *et al.* (2000 ▶). For the stability of the temperature controller used for the data collection, see: Cosier & Glazer (1986 ▶).
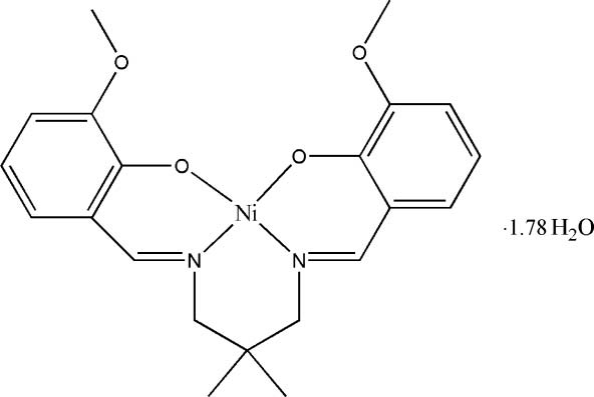


## Experimental

### 

#### Crystal data

[Ni(C_21_H_24_N_2_O_4_)]·1.78H_2_O
*M* *_r_* = 459.29
Monoclinic, 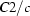

*a* = 23.2513 (6) Å
*b* = 9.2709 (2) Å
*c* = 20.8024 (5) Å
β = 111.291 (1)°
*V* = 4178.12 (17) Å^3^
*Z* = 8
Mo *K*α radiation
μ = 0.97 mm^−1^
*T* = 100 K
0.48 × 0.06 × 0.04 mm

#### Data collection

Bruker SMART APEXII CCD area-detector diffractometer
Absorption correction: multi-scan (**SADABS**; Bruker, 2005 ▶) *T*_min_ = 0.655, *T*_max_ = 0.959
19991 measured reflections
6519 independent reflections
4609 reflections with *I* > 2σ(*I*)
*R*_int_ = 0.040

#### Refinement

*R*[*F* ^2^ > 2σ(*F* ^2^)] = 0.045
*wR*(*F* ^2^) = 0.098
*S* = 1.06
6519 reflections
275 parameters
H-atom parameters constrained
Δρ_max_ = 0.56 e Å^−3^
Δρ_min_ = −0.43 e Å^−3^

### 

Data collection: *APEX2* (Bruker, 2005 ▶); cell refinement: *SAINT* (Bruker, 2005 ▶); data reduction: *SAINT*; program(s) used to solve structure: *SHELXTL* (Sheldrick, 2008 ▶); program(s) used to refine structure: *SHELXTL*; molecular graphics: *SHELXTL*; software used to prepare material for publication: *SHELXTL* and *PLATON* (Spek, 2009 ▶).

## Supplementary Material

Crystal structure: contains datablocks global, I. DOI: 10.1107/S1600536809014500/sj2621sup1.cif

Structure factors: contains datablocks I. DOI: 10.1107/S1600536809014500/sj2621Isup2.hkl

Additional supplementary materials:  crystallographic information; 3D view; checkCIF report

## Figures and Tables

**Table 1:** Hydrogen-bond geometry (Å, °)

*D*—H⋯*A*	*D*—H	H⋯*A*	*D*⋯*A*	*D*—H⋯*A*
O1*W*—H1*W*1⋯O2*W*^i^	0.84	2.08	2.913 (2)	175
O2*W*—H1*W*2⋯O2	0.86	2.54	3.089 (2)	123
O2*W*—H1*W*2⋯O4	0.86	2.10	2.846 (2)	145
O2*W*—H2*W*2⋯O1	0.86	2.22	2.942 (2)	142
O2*W*—H2*W*2⋯O3	0.86	2.16	2.905 (2)	145
O3*W*—H1*W*3⋯O2*W*^i^	0.89	2.11	2.991 (3)	169
C10—H10*B*⋯O2^ii^	0.97	2.48	3.251 (2)	136
C8—H8*B*⋯*Cg*1^iii^	0.97	2.57	3.370 (2)	139
C13—H13*A*⋯*Cg*1^ii^	0.93	2.75	3.377 (2)	125

Symmetry codes: (i) 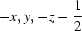; (ii) 
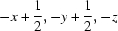
; (iii) 
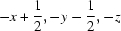
. *Cg*1 is the centroid of the C1–C6 ring.

**Fig. 1.**
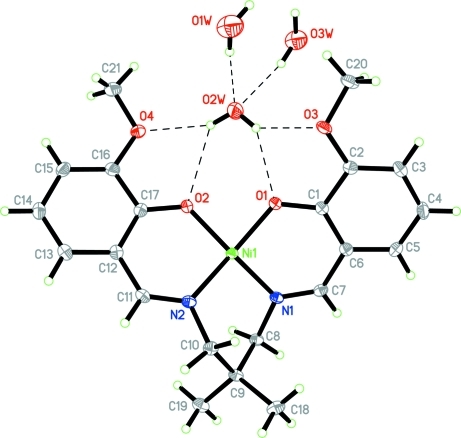
The assymetric unit of the title compound, showing 50% probability displacement ellipsoids and the atomic numbering. Hydrogen bonds are drawn as dashed lines.

**Fig. 2.**
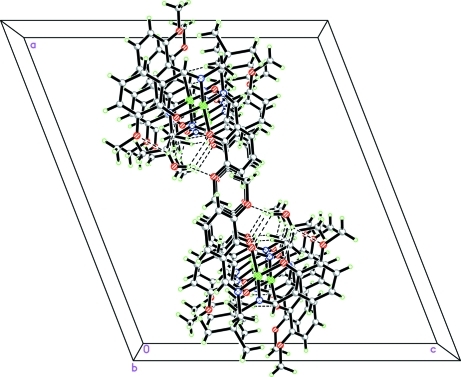
The crystal packing of the title compound viewed down the b-axis, showing 1-dimensional extended chains along the b-axis. Intermolecular interactions are drawn as dashed lines.

## supplementary crystallographic information

### Comment

Schiff base complexes are some of the most important stereochemical models in transition metal coordination chemistry, with their ease of preparation and structural variations (Granovski *et al.*, 1993). Metal derivatives of Schiff bases have been studied extensively, and copper(II) and nickel(II) complexes play a major role in both synthetic and structural research (Elmali *et al.*, 2000; Blower, 1998; Granovski *et al.*, 1993; Li & Chang, 1991; Shahrokhian *et al.*, 2000). Tetradentate Schiff base metal complexes may form *trans* or *cis* planar or tetrahedral structures (Elmali *et al.*, 2000).

In the title compound (Fig. 1), the Ni^II^ ion shows a slightly distorted planar geometry which is coordinated by two imine N atoms and two phenol O atoms of the tetradentate Schiff base ligand. The bond lengths (Allen *et al.*, 1987) and angles are within normal ranges and are comparable with the related structures (Clark *et al.,* 1968, 1969, 1970). The dihedral angle between two benzene rings is 38.84 (9)°.

Of the three solvent water molecules, two of them lie across different crystallographic twofold rotation axes and one of them is partially occupied with a refined occupancy factor of 0.570 (7). The water molecules are linked together as tetramers in *R_2_^2^(8)* ring motifs which also connect two neighbouring molecules of the complex.  The crystal structure is further stabilized by intermolecular C—H···O and C—H···π interactions (Table 1) which link neighbouring molecules into 1-dimensional extended chains along the *b*-axis (Fig. 2).  Other interesting features of the crystal structure are the short intermolecular C1···C8^iii^ [3.204 (3) Å], C1···C11^ii^ [3.364 (3) Å], C2···C8^iii^ [3.365 (3)], C7···O1^iii^ [3.199 (2) Å], and C11···O1^ii^ [3.205 (2) Å] contacts (symmetry operations as in Table 1) which are shorter than the sum of the van der Waals radii of these atoms.

### Experimental

A chloroform solution (40 ml) of *N,N'*-ethylene-bis-(3-methoxy-2- hydroxysalicylaldimine) (1 mmol) was added to a methanol solution (20 ml) of NiCl_2_.6H_2_O (1.05 mmol, 237 mg). The mixture was refluxed for 30 min and then filtered. After keeping the filtrate in air, deep-green needle-shaped crystals were formed at the bottom of the vessel on slow evaporation of the solvent.

### Refinement

The water H-atoms were located from the difference Fourier map and constrained to refine with the parent atom with the U_iso_(H) = 1.5 U_eq_(O). The rest of the hydrogen atoms were positioned geometrically [C—H = 0.93–0.97 Å] and refined using a riding approximation model. A rotating-group model was used for the methyl groups of the methoxy substituents.

### Crystal data

**Table:**

[Ni(C_21_H_24_N_2_O_4_)]·1.78H_2_O	*F*(000) = 1935
*M**_r_* = 459.29	*D*_x_ = 1.460 Mg m^−^^3^
Monoclinic, *C*2/*c*	Mo *K*α radiation, λ = 0.71073 Å
Hall symbol: -C 2yc	Cell parameters from 5799 reflections
*a* = 23.2513 (6) Å	θ = 2.4–30.9°
*b* = 9.2709 (2) Å	µ = 0.97 mm^−^^1^
*c* = 20.8024 (5) Å	*T* = 100 K
β = 111.291 (1)°	Needle, green
*V* = 4178.12 (17) Å^3^	0.48 × 0.06 × 0.04 mm
*Z* = 8	

### Data collection

**Table:**

Bruker SMART APEXII CCD area-detector diffractometer	6519 independent reflections
Radiation source: fine-focus sealed tube	4609 reflections with *I* > 2σ(*I*)
graphite	*R*_int_ = 0.040
φ and ω scans	θ_max_ = 30.9°, θ_min_ = 2.1°
Absorption correction: multi-scan (*SADABS*; Bruker, 2005)	*h* = −33→30
*T*_min_ = 0.655, *T*_max_ = 0.959	*k* = −13→13
19991 measured reflections	*l* = −30→30

### Refinement

**Table:**

Refinement on *F*^2^	Primary atom site location: structure-invariant direct methods
Least-squares matrix: full	Secondary atom site location: difference Fourier map
*R*[*F*^2^ > 2σ(*F*^2^)] = 0.045	Hydrogen site location: inferred from neighbouring sites
*wR*(*F*^2^) = 0.098	H-atom parameters constrained
*S* = 1.06	*w* = 1/[σ^2^(*F*_o_^2^) + (0.0393*P*)^2^ + 1.0555*P*] where *P* = (*F*_o_^2^ + 2*F*_c_^2^)/3
6519 reflections	(Δ/σ)_max_ = 0.001
275 parameters	Δρ_max_ = 0.56 e Å^−^^3^
0 restraints	Δρ_min_ = −0.43 e Å^−^^3^

### Special details

**Table:**

Experimental. The crystal was placed in the cold stream of an Oxford Cyrosystems Cobra open-flow nitrogen cryostat (Cosier & Glazer, 1986) operating at 100.0 (1)K.
Geometry. All esds (except the esd in the dihedral angle between two l.s. planes) are estimated using the full covariance matrix. The cell esds are taken into account individually in the estimation of esds in distances, angles and torsion angles; correlations between esds in cell parameters are only used when they are defined by crystal symmetry. An approximate (isotropic) treatment of cell esds is used for estimating esds involving l.s. planes.
Refinement. Refinement of F^2^ against ALL reflections. The weighted R-factor wR and goodness of fit S are based on F^2^, conventional R-factors R are based on F, with F set to zero for negative F^2^. The threshold expression of F^2^ > 2sigma(F^2^) is used only for calculating R-factors(gt) etc. and is not relevant to the choice of reflections for refinement. R-factors based on F^2^ are statistically about twice as large as those based on F, and R- factors based on ALL data will be even larger.

### Fractional atomic coordinates and isotropic or equivalent isotropic displacement parameters (Å^2^)

**Table:**

	*x*	*y*	*z*	*U*_iso_*/*U*_eq_	Occ. (<1)
Ni1	0.236534 (10)	0.03020 (3)	0.016967 (11)	0.01197 (8)	
O1	0.20185 (6)	−0.04173 (15)	−0.07256 (6)	0.0141 (3)	
O2	0.15706 (6)	0.08978 (16)	0.00363 (6)	0.0146 (3)	
O3	0.15150 (6)	−0.03871 (16)	−0.20732 (7)	0.0198 (3)	
O4	0.03874 (6)	0.13066 (18)	−0.03278 (7)	0.0263 (4)	
N1	0.30718 (7)	−0.08539 (19)	0.04015 (7)	0.0126 (3)	
N2	0.27481 (7)	0.15663 (19)	0.09066 (7)	0.0131 (3)	
C1	0.23268 (8)	−0.1077 (2)	−0.10589 (9)	0.0128 (4)	
C2	0.20584 (8)	−0.1127 (2)	−0.17960 (9)	0.0143 (4)	
C3	0.23389 (9)	−0.1867 (2)	−0.21739 (10)	0.0184 (4)	
H3A	0.2162	−0.1864	−0.2653	0.022*	
C4	0.28894 (9)	−0.2625 (3)	−0.18454 (10)	0.0215 (5)	
H4A	0.3070	−0.3147	−0.2104	0.026*	
C5	0.31593 (9)	−0.2592 (2)	−0.11392 (10)	0.0191 (5)	
H5A	0.3520	−0.3111	−0.0920	0.023*	
C6	0.28970 (8)	−0.1783 (2)	−0.07416 (9)	0.0139 (4)	
C7	0.32108 (8)	−0.1720 (2)	−0.00063 (9)	0.0149 (4)	
H7A	0.3539	−0.2351	0.0191	0.018*	
C8	0.34214 (8)	−0.0980 (2)	0.11514 (9)	0.0143 (4)	
H8A	0.3701	−0.1794	0.1235	0.017*	
H8B	0.3135	−0.1169	0.1384	0.017*	
C9	0.37932 (8)	0.0387 (2)	0.14565 (9)	0.0145 (4)	
C10	0.34240 (8)	0.1725 (2)	0.11110 (9)	0.0151 (4)	
H10A	0.3559	0.2534	0.1426	0.018*	
H10B	0.3517	0.1951	0.0704	0.018*	
C11	0.24663 (8)	0.2501 (2)	0.11465 (9)	0.0136 (4)	
H11A	0.2713	0.3124	0.1484	0.016*	
C12	0.18108 (8)	0.2677 (2)	0.09453 (9)	0.0137 (4)	
C13	0.15822 (9)	0.3744 (2)	0.12736 (9)	0.0165 (4)	
H13A	0.1857	0.4290	0.1627	0.020*	
C14	0.09596 (9)	0.3987 (2)	0.10780 (10)	0.0185 (4)	
H14A	0.0815	0.4692	0.1299	0.022*	
C15	0.05432 (9)	0.3170 (2)	0.05440 (10)	0.0197 (5)	
H15A	0.0121	0.3326	0.0415	0.024*	
C16	0.07557 (9)	0.2138 (2)	0.02094 (10)	0.0176 (4)	
C17	0.14009 (8)	0.1858 (2)	0.03968 (9)	0.0140 (4)	
C18	0.44051 (8)	0.0392 (3)	0.13346 (11)	0.0207 (5)	
H18A	0.4323	0.0397	0.0848	0.031*	
H18B	0.4638	−0.0454	0.1538	0.031*	
H18C	0.4637	0.1237	0.1542	0.031*	
C19	0.39200 (9)	0.0387 (3)	0.22342 (10)	0.0212 (5)	
H19A	0.4149	0.1235	0.2440	0.032*	
H19B	0.4155	−0.0455	0.2441	0.032*	
H19C	0.3535	0.0379	0.2307	0.032*	
C20	0.11522 (10)	−0.0671 (3)	−0.27807 (10)	0.0265 (5)	
H20A	0.0770	−0.0152	−0.2908	0.040*	
H20B	0.1069	−0.1686	−0.2843	0.040*	
H20C	0.1374	−0.0365	−0.3066	0.040*	
C21	−0.02618 (9)	0.1585 (3)	−0.05688 (11)	0.0341 (6)	
H21A	−0.0470	0.0964	−0.0952	0.051*	
H21B	−0.0338	0.2573	−0.0712	0.051*	
H21C	−0.0413	0.1404	−0.0204	0.051*	
O1W	0.0000	0.1551 (3)	−0.2500	0.0449 (7)	
H1W1	−0.0179	0.1045	−0.2849	0.067*	
O2W	0.06628 (7)	−0.03162 (18)	−0.13412 (8)	0.0316 (4)	
H1W2	0.0657	−0.0153	−0.0937	0.047*	
H2W2	0.1022	−0.0323	−0.1371	0.047*	
O3W	0.0000	−0.2322 (4)	−0.2500	0.0320 (14)	0.570 (7)
H1W3	−0.0239	−0.1811	−0.2860	0.048*	0.570 (7)

### Atomic displacement parameters (Å^2^)

**Table:**

	*U*^11^	*U*^22^	*U*^33^	*U*^12^	*U*^13^	*U*^23^
Ni1	0.01173 (11)	0.01265 (14)	0.01018 (11)	0.00058 (11)	0.00237 (8)	−0.00026 (11)
O1	0.0139 (6)	0.0158 (8)	0.0120 (6)	0.0011 (6)	0.0038 (5)	−0.0010 (6)
O2	0.0139 (6)	0.0159 (8)	0.0136 (6)	0.0012 (6)	0.0046 (5)	−0.0023 (6)
O3	0.0219 (7)	0.0210 (9)	0.0112 (6)	0.0054 (7)	−0.0001 (5)	−0.0023 (6)
O4	0.0119 (6)	0.0375 (10)	0.0256 (7)	0.0012 (7)	0.0020 (6)	−0.0132 (8)
N1	0.0132 (7)	0.0132 (9)	0.0104 (7)	−0.0018 (7)	0.0029 (6)	0.0011 (7)
N2	0.0137 (7)	0.0136 (9)	0.0105 (7)	−0.0004 (7)	0.0026 (6)	0.0029 (7)
C1	0.0146 (8)	0.0109 (10)	0.0133 (8)	−0.0031 (8)	0.0057 (7)	0.0002 (8)
C2	0.0155 (8)	0.0113 (11)	0.0150 (8)	−0.0020 (8)	0.0042 (7)	0.0000 (8)
C3	0.0231 (10)	0.0193 (12)	0.0135 (9)	−0.0021 (9)	0.0073 (8)	−0.0020 (9)
C4	0.0229 (10)	0.0242 (13)	0.0209 (10)	−0.0005 (10)	0.0123 (8)	−0.0073 (10)
C5	0.0144 (9)	0.0224 (13)	0.0210 (10)	0.0031 (9)	0.0070 (8)	−0.0004 (10)
C6	0.0142 (8)	0.0127 (10)	0.0150 (8)	−0.0030 (8)	0.0055 (7)	−0.0010 (8)
C7	0.0120 (8)	0.0144 (11)	0.0173 (9)	−0.0009 (8)	0.0042 (7)	0.0008 (9)
C8	0.0145 (8)	0.0146 (11)	0.0116 (8)	0.0000 (8)	0.0023 (7)	0.0028 (8)
C9	0.0140 (8)	0.0145 (11)	0.0132 (8)	−0.0025 (8)	0.0029 (7)	0.0002 (8)
C10	0.0146 (8)	0.0150 (11)	0.0161 (9)	−0.0028 (8)	0.0059 (7)	0.0012 (9)
C11	0.0183 (9)	0.0116 (11)	0.0101 (8)	−0.0026 (8)	0.0044 (7)	0.0020 (8)
C12	0.0175 (9)	0.0120 (11)	0.0129 (8)	0.0001 (8)	0.0071 (7)	0.0030 (8)
C13	0.0226 (9)	0.0129 (11)	0.0149 (9)	−0.0025 (9)	0.0078 (7)	0.0007 (8)
C14	0.0240 (10)	0.0166 (12)	0.0174 (9)	0.0038 (9)	0.0104 (8)	0.0009 (9)
C15	0.0156 (9)	0.0258 (13)	0.0188 (9)	0.0040 (9)	0.0076 (7)	0.0013 (10)
C16	0.0169 (9)	0.0205 (12)	0.0142 (9)	0.0008 (9)	0.0043 (7)	0.0004 (9)
C17	0.0164 (8)	0.0131 (11)	0.0131 (8)	0.0020 (8)	0.0061 (7)	0.0043 (8)
C18	0.0137 (8)	0.0220 (12)	0.0249 (10)	−0.0019 (9)	0.0052 (8)	0.0020 (10)
C19	0.0222 (10)	0.0235 (13)	0.0149 (9)	−0.0025 (10)	0.0033 (7)	−0.0007 (9)
C20	0.0263 (11)	0.0322 (15)	0.0139 (9)	0.0054 (11)	−0.0012 (8)	−0.0034 (10)
C21	0.0135 (9)	0.0545 (18)	0.0291 (11)	0.0012 (11)	0.0016 (8)	−0.0149 (13)
O1W	0.0409 (14)	0.0342 (16)	0.0516 (16)	0.000	0.0072 (12)	0.000
O2W	0.0210 (7)	0.0427 (11)	0.0294 (8)	−0.0017 (8)	0.0073 (6)	−0.0150 (8)
O3W	0.031 (2)	0.023 (3)	0.038 (2)	0.000	0.0070 (18)	0.000

### Geometric parameters (Å, °)

**Table:**

Ni1—O2	1.8505 (13)	C9—C19	1.535 (3)
Ni1—O1	1.8636 (13)	C10—H10A	0.9700
Ni1—N1	1.8719 (16)	C10—H10B	0.9700
Ni1—N2	1.8776 (16)	C11—C12	1.436 (2)
O1—C1	1.315 (2)	C11—H11A	0.9300
O2—C17	1.313 (2)	C12—C13	1.411 (3)
O3—C2	1.368 (2)	C12—C17	1.413 (3)
O3—C20	1.430 (2)	C13—C14	1.373 (3)
O4—C16	1.372 (2)	C13—H13A	0.9300
O4—C21	1.431 (2)	C14—C15	1.402 (3)
N1—C7	1.292 (2)	C14—H14A	0.9300
N1—C8	1.479 (2)	C15—C16	1.376 (3)
N2—C11	1.291 (2)	C15—H15A	0.9300
N2—C10	1.479 (2)	C16—C17	1.430 (2)
C1—C6	1.409 (3)	C18—H18A	0.9600
C1—C2	1.431 (2)	C18—H18B	0.9600
C2—C3	1.373 (3)	C18—H18C	0.9600
C3—C4	1.402 (3)	C19—H19A	0.9600
C3—H3A	0.9300	C19—H19B	0.9600
C4—C5	1.372 (3)	C19—H19C	0.9600
C4—H4A	0.9300	C20—H20A	0.9600
C5—C6	1.409 (3)	C20—H20B	0.9600
C5—H5A	0.9300	C20—H20C	0.9600
C6—C7	1.437 (2)	C21—H21A	0.9600
C7—H7A	0.9300	C21—H21B	0.9600
C8—C9	1.536 (3)	C21—H21C	0.9600
C8—H8A	0.9700	O1W—H1W1	0.8368
C8—H8B	0.9700	O2W—H1W2	0.8598
C9—C10	1.531 (3)	O2W—H2W2	0.8602
C9—C18	1.533 (3)	O3W—H1W3	0.8900
			
O2—Ni1—O1	84.88 (5)	C9—C10—H10A	108.7
O2—Ni1—N1	160.84 (7)	N2—C10—H10B	108.7
O1—Ni1—N1	94.21 (6)	C9—C10—H10B	108.7
O2—Ni1—N2	95.02 (6)	H10A—C10—H10B	107.6
O1—Ni1—N2	160.77 (7)	N2—C11—C12	126.70 (18)
N1—Ni1—N2	92.04 (7)	N2—C11—H11A	116.7
C1—O1—Ni1	124.91 (11)	C12—C11—H11A	116.7
C17—O2—Ni1	127.39 (12)	C13—C12—C17	120.35 (17)
C2—O3—C20	116.80 (15)	C13—C12—C11	119.01 (18)
C16—O4—C21	116.83 (17)	C17—C12—C11	120.51 (17)
C7—N1—C8	118.09 (16)	C14—C13—C12	120.92 (18)
C7—N1—Ni1	126.32 (13)	C14—C13—H13A	119.5
C8—N1—Ni1	114.32 (12)	C12—C13—H13A	119.5
C11—N2—C10	117.23 (16)	C13—C14—C15	119.78 (19)
C11—N2—Ni1	125.31 (13)	C13—C14—H14A	120.1
C10—N2—Ni1	115.85 (12)	C15—C14—H14A	120.1
O1—C1—C6	124.71 (16)	C16—C15—C14	120.34 (18)
O1—C1—C2	118.08 (16)	C16—C15—H15A	119.8
C6—C1—C2	117.18 (17)	C14—C15—H15A	119.8
O3—C2—C3	124.56 (17)	O4—C16—C15	124.75 (17)
O3—C2—C1	114.34 (16)	O4—C16—C17	113.76 (17)
C3—C2—C1	121.10 (18)	C15—C16—C17	121.48 (18)
C2—C3—C4	120.73 (18)	O2—C17—C12	124.81 (17)
C2—C3—H3A	119.6	O2—C17—C16	118.05 (17)
C4—C3—H3A	119.6	C12—C17—C16	117.11 (18)
C5—C4—C3	119.49 (18)	C9—C18—H18A	109.5
C5—C4—H4A	120.3	C9—C18—H18B	109.5
C3—C4—H4A	120.3	H18A—C18—H18B	109.5
C4—C5—C6	120.88 (18)	C9—C18—H18C	109.5
C4—C5—H5A	119.6	H18A—C18—H18C	109.5
C6—C5—H5A	119.6	H18B—C18—H18C	109.5
C5—C6—C1	120.43 (17)	C9—C19—H19A	109.5
C5—C6—C7	119.07 (17)	C9—C19—H19B	109.5
C1—C6—C7	120.48 (17)	H19A—C19—H19B	109.5
N1—C7—C6	124.96 (18)	C9—C19—H19C	109.5
N1—C7—H7A	117.5	H19A—C19—H19C	109.5
C6—C7—H7A	117.5	H19B—C19—H19C	109.5
N1—C8—C9	112.44 (16)	O3—C20—H20A	109.5
N1—C8—H8A	109.1	O3—C20—H20B	109.5
C9—C8—H8A	109.1	H20A—C20—H20B	109.5
N1—C8—H8B	109.1	O3—C20—H20C	109.5
C9—C8—H8B	109.1	H20A—C20—H20C	109.5
H8A—C8—H8B	107.8	H20B—C20—H20C	109.5
C10—C9—C18	108.41 (16)	O4—C21—H21A	109.5
C10—C9—C19	110.81 (16)	O4—C21—H21B	109.5
C18—C9—C19	109.85 (15)	H21A—C21—H21B	109.5
C10—C9—C8	109.82 (15)	O4—C21—H21C	109.5
C18—C9—C8	110.55 (17)	H21A—C21—H21C	109.5
C19—C9—C8	107.40 (16)	H21B—C21—H21C	109.5
N2—C10—C9	114.23 (16)	H1W2—O2W—H2W2	115.6
N2—C10—H10A	108.7		
			
O2—Ni1—O1—C1	179.08 (15)	C8—N1—C7—C6	176.88 (17)
N1—Ni1—O1—C1	−20.13 (16)	Ni1—N1—C7—C6	10.6 (3)
N2—Ni1—O1—C1	88.5 (2)	C5—C6—C7—N1	168.97 (19)
O1—Ni1—O2—C17	−162.95 (16)	C1—C6—C7—N1	−12.7 (3)
N1—Ni1—O2—C17	109.0 (2)	C7—N1—C8—C9	116.78 (19)
N2—Ni1—O2—C17	−2.25 (16)	Ni1—N1—C8—C9	−75.31 (16)
O2—Ni1—N1—C7	90.5 (2)	N1—C8—C9—C10	37.6 (2)
O1—Ni1—N1—C7	3.93 (17)	N1—C8—C9—C18	−81.95 (19)
N2—Ni1—N1—C7	−157.87 (17)	N1—C8—C9—C19	158.22 (15)
O2—Ni1—N1—C8	−76.3 (2)	C11—N2—C10—C9	123.82 (18)
O1—Ni1—N1—C8	−162.81 (13)	Ni1—N2—C10—C9	−69.85 (17)
N2—Ni1—N1—C8	35.38 (13)	C18—C9—C10—N2	153.78 (16)
O2—Ni1—N2—C11	−2.15 (16)	C19—C9—C10—N2	−85.59 (19)
O1—Ni1—N2—C11	86.7 (2)	C8—C9—C10—N2	32.9 (2)
N1—Ni1—N2—C11	−164.32 (16)	C10—N2—C11—C12	170.83 (17)
O2—Ni1—N2—C10	−167.23 (13)	Ni1—N2—C11—C12	5.9 (3)
O1—Ni1—N2—C10	−78.4 (2)	N2—C11—C12—C13	178.81 (18)
N1—Ni1—N2—C10	30.60 (13)	N2—C11—C12—C17	−5.2 (3)
Ni1—O1—C1—C6	23.2 (3)	C17—C12—C13—C14	1.4 (3)
Ni1—O1—C1—C2	−158.92 (14)	C11—C12—C13—C14	177.37 (18)
C20—O3—C2—C3	14.6 (3)	C12—C13—C14—C15	−0.2 (3)
C20—O3—C2—C1	−165.57 (18)	C13—C14—C15—C16	−0.8 (3)
O1—C1—C2—O3	3.8 (3)	C21—O4—C16—C15	2.5 (3)
C6—C1—C2—O3	−178.18 (17)	C21—O4—C16—C17	−176.59 (19)
O1—C1—C2—C3	−176.36 (18)	C14—C15—C16—O4	−178.51 (19)
C6—C1—C2—C3	1.7 (3)	C14—C15—C16—C17	0.6 (3)
O3—C2—C3—C4	−178.48 (19)	Ni1—O2—C17—C12	3.2 (3)
C1—C2—C3—C4	1.6 (3)	Ni1—O2—C17—C16	−178.97 (13)
C2—C3—C4—C5	−1.9 (3)	C13—C12—C17—O2	176.22 (18)
C3—C4—C5—C6	−1.3 (3)	C11—C12—C17—O2	0.3 (3)
C4—C5—C6—C1	4.7 (3)	C13—C12—C17—C16	−1.6 (3)
C4—C5—C6—C7	−176.9 (2)	C11—C12—C17—C16	−177.50 (18)
O1—C1—C6—C5	173.11 (18)	O4—C16—C17—O2	1.8 (3)
C2—C1—C6—C5	−4.8 (3)	C15—C16—C17—O2	−177.33 (18)
O1—C1—C6—C7	−5.2 (3)	O4—C16—C17—C12	179.80 (17)
C2—C1—C6—C7	176.85 (18)	C15—C16—C17—C12	0.6 (3)

### Hydrogen-bond geometry (Å, °)

**Table:**

*D*—H···*A*	*D*—H	H···*A*	*D*···*A*	*D*—H···*A*
O1W—H1W1···O2W^i^	0.84	2.08	2.913 (2)	175
O2W—H1W2···O2	0.86	2.54	3.089 (2)	123
O2W—H1W2···O4	0.86	2.10	2.846 (2)	145
O2W—H2W2···O1	0.86	2.22	2.942 (2)	142
O2W—H2W2···O3	0.86	2.16	2.905 (2)	145
O3W—H1W3···O2W^i^	0.89	2.11	2.991 (3)	169
C10—H10B···O2^ii^	0.97	2.48	3.251 (2)	136
C8—H8B···Cg1^iii^	0.97	2.57	3.370 (2)	139
C13—H13A···Cg1^ii^	0.93	2.75	3.377 (2)	125

Symmetry codes: (i) −*x*, *y*, −*z*−1/2; (ii) −*x*+1/2, −*y*+1/2, −*z*; (iii) −*x*+1/2, −*y*−1/2, −*z*.
